# Neurofilament light chain: defining the analyte

**DOI:** 10.1093/braincomms/fcac070

**Published:** 2022-03-21

**Authors:** Claire A. Leckey, Henrik Zetterberg

**Affiliations:** 1 UK Dementia Research Institute at UCL, London, UK; 2 Department of Neurodegenerative Disease, UCL Institute of Neurology, London, UK; 3 Centre for Translational Omics, UCL Great Ormond Street Institute of Child Health, London, UK; 4 Department of Psychiatry and Neurochemistry, Institute of Neuroscience and Physiology, Sahlgrenska Academy at University of Gothenburg, Mölndal, Sweden; 5 Clinical Neurochemistry Laboratory, Sahlgrenska University Hospital, Mölndal, Sweden; 6 Hong Kong Center for Neurodegenerative Diseases, Clear Water Bay, Hong Kong, China

## Abstract

This scientific commentary refers to ‘A map of neurofilament light chain species in brain and cerebrospinal fluid and alterations in Alzheimer’s disease’ by Budelier *et al*. (https://doi.org/10.1093/braincomms/fcac045).

**This scientific commentary refers to ‘A map of neurofilament light chain species in brain and cerebrospinal fluid and alterations in Alzheimer’s disease’ by Budelier *et al*. (https://doi.org/10.1093/braincomms/fcac045).**

In 1996, Lars Rosengren,^1^ a histologist and neurologist at Sahlgrenska University Hospital, Gothenburg, Sweden, published the first enzyme-linked immunosorbent assay (ELISA) to measure neurofilament light chain (NfL) concentration in human cerebrospinal fluid (CSF) using polyclonal antibodies. He and his team reported higher NfL concentration in CSF samples from patients with amyotrophic lateral sclerosis and Alzheimer's disease compared with controls. Pilot data on increased CSF NfL concentration in vascular dementia, olivopontocerebellar atrophy, normal pressure hydrocephalus, cerebral infarctions and multiple sclerosis were also presented.^1^ Since then, NfL has emerged as an intriguing and clinically meaningful fluid-based biomarker of neuronal axonal injury and degeneration that is elevated in multiple neurodegenerative diseases,^2^ as well as in neuroinflammation,^3^ CNS infections^4^ and acute brain injury.^5^ Currently, these studies have focused on its quantitation by immunoassay-based methods, which have offered the analytical sensitivity required to measure and compare NfL levels in CSF (by ELISA), as well as plasma and serum (by Single molecule array or Meso Scale Discovery technology), across various neurodegenerative disease cohorts. Remarkably, characterization of the exact species of NfL that are present in CSF and being measured has remained a pressing question that has been left unanswered.

Given the clinical utility of the marker, as well as its use in clinical trials to detect disease-modifying effects of novel treatments against brain diseases, standardizing NfL assays to each other would be valuable. To this end, certified reference materials that have been value-assigned using certified reference methods are needed. However, a pre-requisite for this type of work is detailed knowledge on the exact form of the analyte to be measured.

In this issue of *Brain Communications*, Budelier and colleagues^6^ present the development and validation of a hybrid immunoprecipitation–mass spectrometry (IP-MS) method combined with tryptic digestion to characterize and quantify NfL in brain tissue and CSF. Using 23 custom antibodies generated against different domains of the full protein sequence, the authors initially identified NfL fragments in brain tissue and CSF pools, whereafter a quantitative assay using three antibodies and isotope-labelled standard peptides was developed.

In the brain, a full-length and a C-terminal fragment of NfL were identified. In CSF, there were at least three major forms of NfL: two rod domain-containing fragments (amino acids 92–224 with some variation at the C-terminus, and amino acids 324–360), as well as a C-terminal fragment containing the tail of NfL (from amino acid 530 to at least 540). No N-terminal fragments were recovered, and full-length NfL was not detectable. These newly identified CSF NfL species were confirmed in a discovery cohort of controls and Alzheimer’s disease participants (*n* = 10), before further validating these findings in a confirmation cohort of participants with Alzheimer’s disease dementia, non-Alzheimer’s disease dementia and healthy controls (*n* = 81).

In agreement with previous studies using immunoassays, Budelier and colleagues showed that NfL was increased in CSF from individuals with Alzheimer’s disease (symptomatic, amyloid-positive) compared with controls (asymptomatic, amyloid-negative). They further demonstrated that the fold change in NfL observed between groups varied depending on the NfL fragment measured, which is a very important observation for projects aimed at developing clinical-grade assays for the biomarker. The highest performing fragments were GMNEALEK (amino acids 324–331) and VEGAGEEQAAK (amino acids 530–540). In particular, the GMNEALEK peptide from the rod domain was found to correlate the best with NfL concentrations derived using the most commonly used commercial ELISA (UmanDiagnostics), suggesting this specific region-targeted IP-MS assay shows the greatest promise as a candidate reference method for CSF NfL.

Additionally, the full assay, in which multiple forms of NfL can be simultaneously quantified, will be invaluable in experimental and human studies examining NfL biology and kinetics, mechanisms of release and turnover, and potential disease-specific changes. Such studies are likely to further benefit from the general conservation of the NfL sequence across animal species, thus requiring minimal to no analytical adaption of the assay for various experimental models.

## Data Availability

Data sharing is not applicable to this article as no new data were created or analysed.
